# Metastatic Recurrence in a Pancreatic Cancer Patient Derived Orthotopic Xenograft (PDOX) Nude Mouse Model Is Inhibited by Neoadjuvant Chemotherapy in Combination with Fluorescence-Guided Surgery with an Anti-CA 19-9-Conjugated Fluorophore

**DOI:** 10.1371/journal.pone.0114310

**Published:** 2014-12-02

**Authors:** Yukihiko Hiroshima, Ali Maawy, Yong Zhang, Takashi Murakami, Masashi Momiyama, Ryutaro Mori, Ryusei Matsuyama, Matthew H. G. Katz, Jason B. Fleming, Takashi Chishima, Kuniya Tanaka, Yasushi Ichikawa, Itaru Endo, Robert M. Hoffman, Michael Bouvet

**Affiliations:** 1 AntiCancer, Inc., San Diego, California, United States of America; 2 Department of Surgery, University of California San Diego, San Diego, California, United States of America; 3 Yokohama City University Graduate School of Medicine, Yokohama, Japan; 4 Department of Surgery, MD Anderson Cancer Center, Houston, Texas, United States of America; National Cancer Institute, United States of America

## Abstract

The aim of this study is to determine the efficacy of neoadjuvant chemotherapy (NAC) with gemcitabine (GEM) in combination with fluorescence-guided surgery (FGS) on a pancreatic cancer patient derived orthotopic xenograft (PDOX) model. A PDOX model was established from a CA19-9-positive, CEA-negative tumor from a patient who had undergone a pancreaticoduodenectomy for pancreatic adenocarcinoma. Mice were randomized to 4 groups: bright light surgery (BLS) only; BLS+NAC; FGS only; and FGS+NAC. An anti-CA19-9 or anti-CEA antibody conjugated to DyLight 650 was administered intravenously via the tail vein of mice with the pancreatic cancer PDOX 24 hours before surgery. The PDOX was brightly labeled with fluorophore-conjugated anti-CA19-9, but not with a fluorophore-conjugated anti-CEA antibody. FGS was performed using the fluorophore-conjugated anti-CA19-9 antibody. FGS had no benefit over BLS to prevent metastatic recurrence. NAC in combination with BLS did not convey an advantage over BLS to prevent metastatic recurrence. However, FGS+NAC significantly reduced the metastatic recurrence frequency to one of 8 mice, compared to FGS only after which metastasis recurred in 6 out of 8 mice, and BLS+NAC with metastatic recurrence in 7 out of 8 mice (p = 0.041). Thus NAC in combination with FGS can reduce or even eliminate metastatic recurrence of pancreatic cancer sensitive to NAC. The present study further emphasizes the power of the PDOX model which enables metastasis to occur and thereby identify the efficacy of NAC in combination with FGS on metastatic recurrence.

## Introduction

Complete tumor resection improves overall survival of pancreatic cancer patients, which is presently 5% at five years [1]. Metastatic relapse often occurs following attempted curative resection of the primary tumor as a result of invisible microscopic tumor deposits left behind. Making tumors fluoresce offers great advantages for tumor detection during surgery in order to achieve complete resection [2], [3]. We have previously shown that fluorescence-guided surgery (FGS) for pancreatic cancer decreased the residual tumor burden and improved overall and disease-free survival in mouse models using fluorescently-labeled human pancreatic cancer cell lines [4]–[6].

Patient-derived orthotopic xenografts (PDOX) recapitulate the biological characteristics of the disease of origin, including metastases [7]–[11] and are a clinically-relevant model for fluorescence-guided surgery [4], [12]–[14].

Recently, many studies reported positive outcomes with neoadjuvant chemotherapy (NAC) of pancreatic cancer [15]–[17]. NAC allows for the identification of those patients with rapidly progressive metastatic disease at the time of preoperative restaging, and can increase the R0 resection rate and reduce the risk of local tumor recurrence [15]. However, a significant number of patients still develop recurrent disease immediately after NAC treatment and subsequent surgical resection [16]–[18]. Therefore, new strategies in addition to NAC are needed to reduce the recurrence of pancreatic cancer. In this study, we determined the efficacy of CA19-9 conjugated with a fluorescent dye to illuminate pancreatic cancer PDOXs for FGS in combination with NAC.

## Materials and Methods

### Animals

Athymic *nu/nu* nude mice (AntiCancer Inc., San Diego, CA), 4–6 weeks old, were used in this study. Mice were kept in a barrier facility under HEPA filtration. Mice were fed with an autoclaved laboratory rodent diet. All mouse surgical procedures and imaging were performed with the animals anesthetized by intramuscular injection of 50% ketamine, 38% xylazine, and 12% acepromazine maleate (0.02 ml). Animals received buprenorphine (0.10 mg/kg ip) immediately prior to surgery and once a day over the next 3 days to ameliorate pain. CO_2_ inhalation was used for euthanasia of all animals at 90 days after surgery. To ensure death following CO_2_ asphyxiation, cervical dislocation was performed. All animal studies were conducted with an AntiCancer, Inc. Institutional Animal Care and Use Committee (IACUC)-protocol specifically approved for this study and in accordance with the principals and procedures outlined in the National Institute of Health Guide for the Care and Use of Animals under Assurance Number A3873-1.

### Establishment of patient derived orthotopic xenograft (PDOX) of pancreatic cancer

Pancreatic cancer patient tumor tissues were obtained at surgery and cut into fragments (3-mm^3^) and transplanted subcutaneously in nude mice [7], [19]. The subcutaneous tumors were then passaged in nude mice both orthotopically and subcutaneously. All patients provided written informed consent and samples were procured and initially transplanted in NOD/SCID under the approval of the Institutional Review Board of MD Anderson Cancer Center.

### Orthotopic tumor implantation

A small 6- to 10-mm transverse incision was made on the left flank of the mouse through the skin and peritoneum. The tail of the pancreas was exposed through this incision, and a single 3-mm^3^ tumor fragment from subcutaneous tumors was sutured to the tail of the pancreas using 8-0 nylon surgical sutures (Ethilon; Ethicon Inc., NJ, USA). On completion, the tail of the pancreas was returned to the abdomen, and the incision was closed in one layer using 6-0 nylon surgical sutures (Ethilon) [7], [20].

### Antibody conjugation and tumor labeling

Monoclonal antibodies specific for carbohydrate antigen 19-9 (CA19-9) and carcinoembryonic antigen (CEA) were obtained from Abcam Inc. (Cambridge, MA, USA) and (Aragen Bioscience, Inc. (Morgan Hill, CA, USA), respectively. The antibodies were labeled with the DyLight 650 Protein Labeling Kit (Thermofisher Scientific, Waltham, MA, USA) according to the manufacturer’s instructions [5], [12], [21]. To determine if the anti-CA19-9 antibody, conjugated with DyLight 650 (anti-CA19-9-650), and the anti-CEA antibody, conjugated with DyLight650 (anti-CEA-650), could label the pancreatic tumor in vivo, 50 µg of anti-CA19-9-650 or anti-CEA-650 were injected into the tail vein of the mice with subcutaneous pancreatic tumors. Twenty-four hours later, whole body images were obtained with the OV100 Small Animal Variable Magnification Imaging System (Olympus, Tokyo, Japan).

### Neoadjuvant chemotherapy

After confirmation of tumor engraftment, 32 mice were randomized to 4 groups; BLS only; BLS+NAC; FGS only; and FGS+NAC. Each treatment arm involved 8 tumor-bearing mice. The mice randomized to NAC-treatment were administered 80 mg/kg gemcitabine (GEM) (Eli Lilly and Company, Indianapolis, IN, USA). GEM was injected i.p. on day 8, 15 and 22. No significant effects on body weight, morbidity, or severe toxicity were observed in NAC-treated mice.

### Fluorescence-guided surgery

For fluorescence-guided surgery (FGS), a 15-mm transverse incision was made on the left flank of the mouse through the skin and peritoneum which was kept open with a retractor. The tail of the pancreas was exposed through this incision. Fifty µg of anti-CA19-9 antibody, conjugated to DyLight 650, was injected via the tail vein in the mice in the FGS group 24 hours before surgery. A MINI MAGLITE LED PRO flashlight (MAG INSTRUMENT, Ontario, CA, USA) coupled to an excitation filter (ET 640/30X, Chroma Technology Corporation, Bellows Falls, VT, USA) was used as the excitation light source. A Canon EOS 60D digital camera with an EF–S18–55 IS lens (Canon, Tokyo, Japan) coupled with an emission filter (HQ700/75M-HCAR, Chroma Technology Corporation) was used as the real-time image capturing device for FGS. BLS was performed under standard bright-field using an MVX10 microscope (Olympus, Tokyo, Japan). After completion of surgery, the incision was closed in one layer using 6-0 nylon surgical sutures, and the mice were allowed to recover in their cages.

### Tissue histology

Tumor samples were removed with surrounding normal tissues at the time of resection. Fresh tissue samples were fixed in 10% formalin and embedded in paraffin before sectioning and staining. Tissue sections (3 µm) were deparaffinized in xylene and rehydrated in an ethanol series. Hematoxylin and eosin (H & E) staining was performed according to standard protocols. For immunohistochemistry, the sections were then treated for 30 min with 0.3% hydrogen peroxide to block endogenous peroxidase activity. The sections were subsequently washed with PBS and unmasked in citrate antigen unmasking solution (Mitsubishi Kagaku Iatron, Inc., Tokyo, Japan) in a water bath for 40 min at 98°C. After incubation with 10% normal goat serum, the sections were incubated with anti-CA19-9 antibody (1∶100) and anti-CEA antibody (1∶100) at 4°C overnight. The bound primary antibodies were detected by binding with an anti-mouse secondary antibody and an avidin/biotin/horseradish peroxidase complex (DAKO Cytomation, Kyoto, Japan) for 30 min at room temperature. The labeled antigens were visualized with a DAB kit (DAKO Cytomation). The sections were counterstained with hematoxylin and observed with a BH-2 microscope (Olympus, Tokyo, Japan) equipped with an INFINITY1 2.0 megapixel CMOS digital camera (Lumenera Corporation, Ottawa, Canada). All images were acquired using INFINITY ANALYZE software (Lumenera Corporation) without post-acquisition processing.

### Evaluation of histopathological response to NAC

Histopathological response to chemotherapy drugs was defined according to Evans’s grading scheme: Grade I, little (<10%) or no tumor cell destruction is evident; Grade IIa, destruction of 10%–50% of tumor cells; Grade IIb, destruction of 51%–90% of tumor cells; Grade III, few (<10%) viable-appearing tumor cells are present; Grade IV, no viable tumor cells are present [22].

### Evaluation of tumor recurrence and progression

To assess for recurrence postoperatively, animals underwent laparotomy 12 weeks after surgery, and the tumors were imaged with the Canon EOS 60D digital camera with an EF–S18–55 IS lens (Canon), excised, harvested and weighed for analysis.

### Statistical analysis

PASWStatistics 18.0 (SPSS, Inc.) was used for statistical analyses. Tumor weight was expressed as mean ± SD. The two-tailed Student’s *t*-test was used to compare continuous variables between 2 groups. Comparisons between categorical variables were analyzed with Fisher’s exact test. A p value <0.05 was considered statistically significant for all comparisons.

## Results

### Antibody labeling

**Figure 1:**
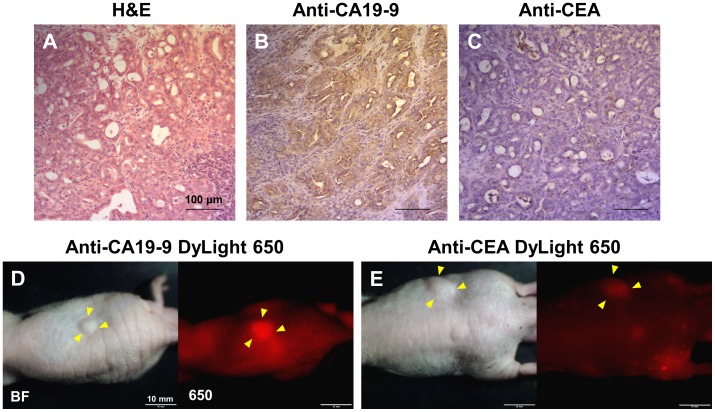
Antibody labelling of the pancreatic cancer patient derived orthotopic xenograft. The patient’s pancreatic cancer was diagnosed as moderately differentiated adenocarcinoma with H&E staining (A). The tumor was strongly stained with anti-CA19-9 antibody (B), whereas the signal was very weak with anti-CEA antibody (C). Scale bars: 100 µm. (D and E) Whole body images of a subcutaneous tumor in nude mice labeled with anti-CA19-9- or anti-CEA-conjugated DyLight 650. Fifty µg anti-CA19-9 DyLight 650 or anti-CEA DyLight 650 was injected in the tail vain of the mice with subcutaneous tumors. Twenty-four hours later, whole body images were taken with the OV100 (Olympus). Yellow arrowheads indicate subcutaneous tumors. The subcutaneous tumors were brightly labeled with anti-CA19-9 DyLight 650 (D), whereas, the fluorescence signal from the tumor labeled with anti-CEA DyLight 650 was very weak (E). Scale bars: 10 mm.

The pancreatic PDOX tumor was diagnosed as moderately differentiated adenocarcinoma with H&E staining (Figure 1A). Based on immunohistochemistry, the PDOX tumor was found to be CA19-9-positive and CEA-negative (Figures 1B and 1C). The PDOX was brightly labeled with anti-CA19-9-650 (Figure 1D), but the fluorescence signal with anti-CEA-650 was very weak (Figure 1E). The fluorescence results were consistent with the immunohistochemical results, and based on them, it was decided to use anti-CA19-9-650 to label the PDOX for FGS. Anti-CA19-9-650 was injected in the tail vein of the mice with PDOX tumors 24 hours before FGS.

### Sensitivity of PDOX to NAC

**Figure 2:**
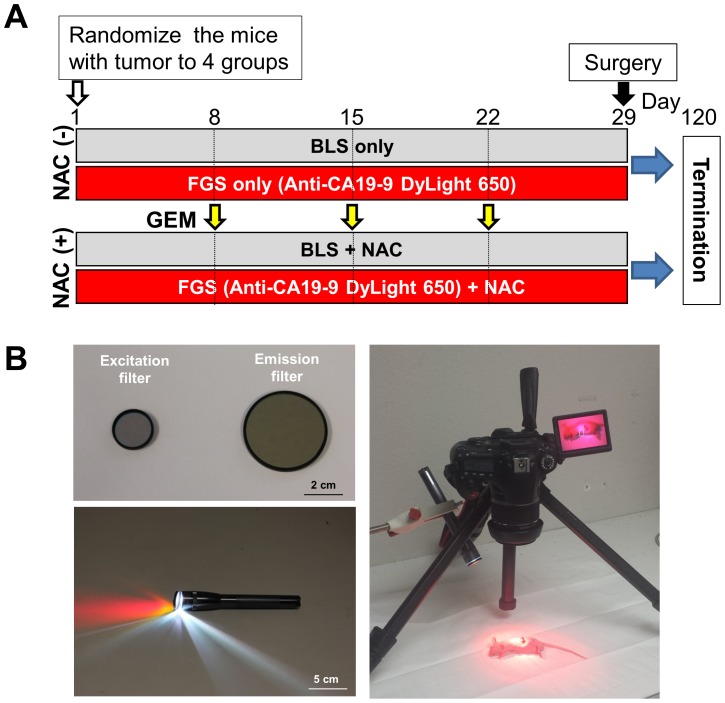
Experimental schema and FGS imaging system. (A) Schema of the experimental design. After confirmation of tumor growth, the PDOXs were randomized to 4 groups: BLS only; BLS+NAC; FGS only; or FGS+NAC. Each treatment arm involved 8 tumor-bearing mice. The mice randomized to the NAC groups were treated with GEM (80 mg/kg) on day 8, 15 and 22. All animals underwent surgery on day 29. BLS was performed under standard bright-field using the MVX10 microscope. Fifty µg of anti-CA19-9 antibody conjugated with DyLight 650 was injected in the tail vain of mice with tumors in the FGS group 24 hours before surgery. FGS was performed using the MINI MAGLITE LED PRO flash light (MAG INSTRUMENT, Ontario, CA, USA) with excitation filter ET640/30X (Chroma Technology Corporation, Bellows Falls, VT, USA) and a Canon EOS 60D digital camera with an EF–S18–55 IS lens (Canon, Tokyo, Japan) and emission filter HQ700/75M-HCAR (Chroma Technology Corporation) under fluorescence navigation (B). Twelve weeks after surgery, animals underwent laparotomy, and the tumors were imaged, weighed and harvested for analysis. Scale bars: 2 cm (filters) and 5 cm (flash light).

**Figure 3:**
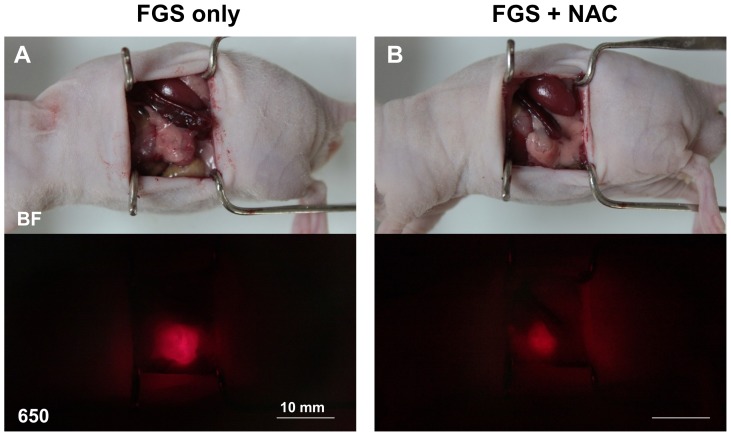
Representative images during FGS with or without NAC. Upper panels indicate bright field (BF) images and lower panels indicate fluorescence images for DyLight 650 (650). The fluorescence in the tumors treated with GEM (B) decreased compared to untreated tumors (A), but were still clearly detected. Scale bars: 10 mm.

**Figure 4:**
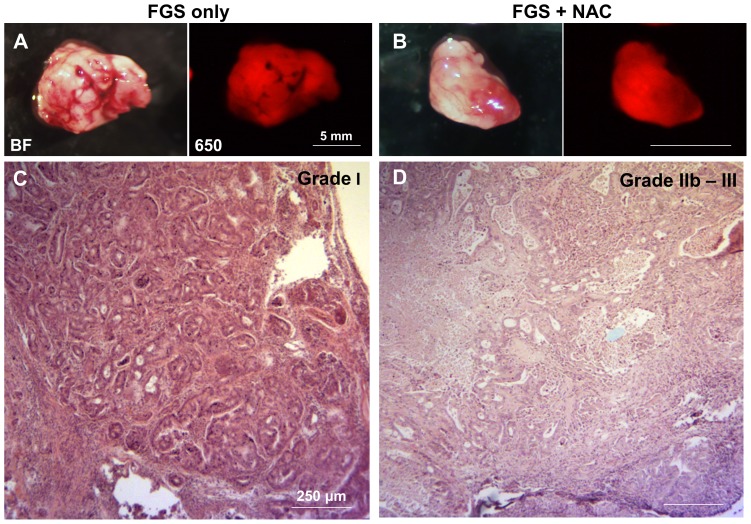
Representative gross and histological images of excised tumors in each treatment group. Left panels of (A) and (B) indicate bright field (BF) images and right panels indicate fluorescence images for DyLight 650 (650). Histopathological response to GEM treatment was defined according to Evans’s grading scheme. The tumors without GEM treatment (FGS only) were comprised of viable cancer cells that formed glandular structures and judged as Grade I (C). In the tumors with GEM treatment, over 50% of cancer cells were dead and replaced by necrotic tissue or stromal cells (D). Treatment efficacy of GEM on the pancreatic cancer PDOX was judged as grade IIb - III (D). Fluorescence decreased in some areas of the tumor treated with GEM, but was sufficient for FGS (B). Scale bars: 5 mm (A and B), 250 µm (C and D).

The PDOX mice were randomized to 4 groups; BLS only; BLS+NAC; FGS only; FGS+NAC. Each treatment arm involved 8 PDOX mice. The mice randomized to the NAC group were treated with GEM on days 8, 15 and 22 (Figure 2). All animals underwent surgery on day 29 (Figures 3). The average excised PDOX tumor weight was 188.5±53.1 mg for BLS-only; 84.5±51.6 mg for BLS+NAC; 299.0±86.3 mg for FGS-only; and 141.8±48.9 mg for FGS+NAC. The average excised tumor weight in the BLS+NAC mice was significantly less than in the BLS-only mice (p = 0.001). The average excised tumor weight in the FGS+NAC mice was also significantly less than FGS-only mice (p<0.001). Upon histological examination, over 50% of cancer cells were dead and replaced by necrotic tissue or stromal cells in the PDOX tumor treated with FGS+NAC and was judged as Evan’s grade IIb - III (Figure 4).

### Effect of NAC on tumor recurrence with BLS or FGS

**Figure 5:**
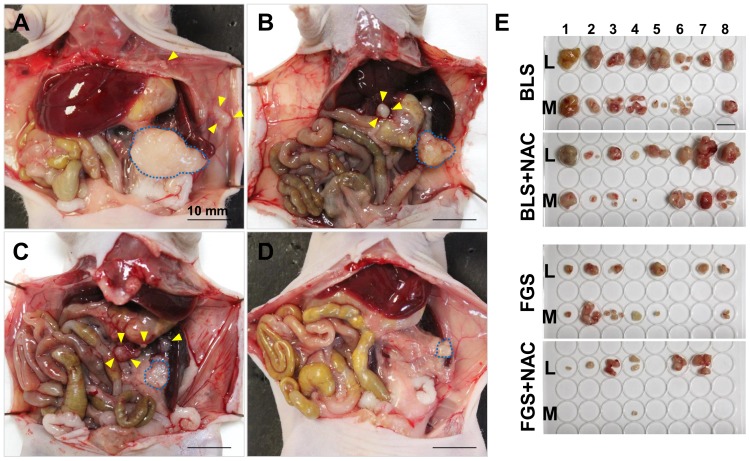
Representative images of the recurrent PDOX tumor. (A) A large local recurrent tumor (surrounded by the blue broken line) and peritoneal dissemination (yellow arrowheads) in a BLS-only treated mouse. (B) A locally recurrent tumor (surrounded by a blue dashed line) and a lymph-node metastasis in the hepatoduodenal ligament (yellow arrow heads) in a BLS+NAC-treated mouse. (C) A small local recurrent tumor (surrounded by a blue dashed line) and peritoneal dissemination (yellow arrow heads) in an FGS-only treated mouse. (D) A small local recurrent tumor (surrounded by a blue dashed line) without metastasis in an FGS+NAC-treated mouse. (E) Gross images of all excised recurrent tumors. Upper lines indicate local recurrent tumors and lower lines indicate metastatic recurrent tumors. The recurrent tumor volume in the FGS group was smaller than in the BLS group. The metastatic recurrent tumor volume in the one FGS+NAC-treated mouse that had metastatic recurrence was smaller than recurrent metastases in the FGS-only treated mice. Scale bars: 10 mm.

**Figure 6:**
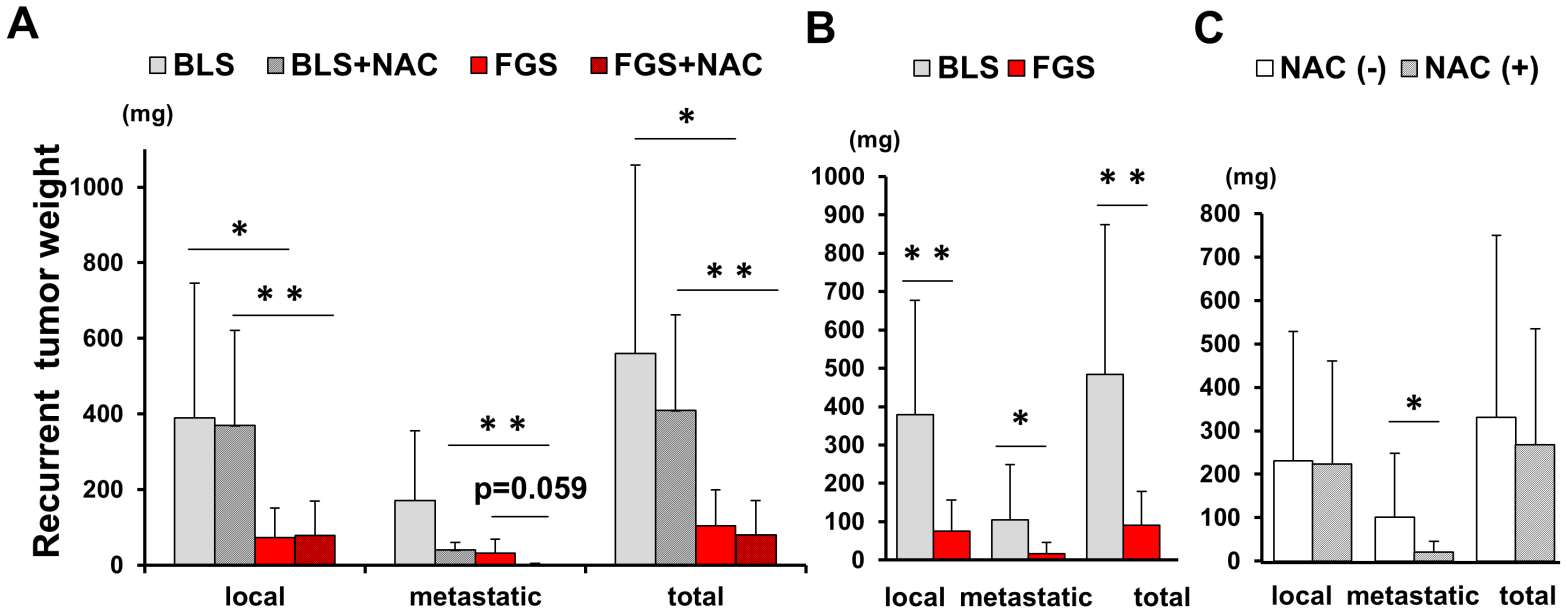
Recurrent tumor weights for each experimental group. (A) Recurrent tumor weight in the BLS-only; BLS+NAC; FGS-only; and FGS+NAC treatment groups. The average local recurrent tumor weight for FGS-only treatment was significantly less than for BLS-only treatment (p = 0.0041). The average local recurrent tumor weight for FGS+NAC treatment was also significantly less than for BLS+NAC treatment (p = 0.008). The average metastatic recurrent tumor weight for FGS+NAC treatment was significantly less than for BLS+NAC treatment (p = 0.001). FGS+NAC treatment reduced recurrence rate significantly compared to FGS-only treatment (p = 0.041) (Table 1). FGS+NAC treatment tended to reduce the metastatic recurrent tumor weight compared to FGS-only treatment (p = 0.059).

With regard to the recurrent tumor weight, the average local recurrent tumor weight was 389.2±356.6 mg in BLS-only treated mice; 369.1±251.9 mg in BLS+NAC-treated mice; 73.0±77.2 mg in FGS-only treated mice; and 78.4±90.8 mg in FGS+NAC-treated mice. The average local recurrent tumor weight in FGS-only treated mice was significantly less than in BLS-only treated mice (p = 0.041). The average metastatic recurrent tumor weight of the pancreatic cancer PDOX was 170.7±184.2 mg for BLS-only treated mice; 40.0±19.7 mg for BLS+NAC-treated mice; 31.3±37.6 mg for FGS-only mice; and 1.3±3.7 mg for FGS+NAC-treated mice. The average metastatic recurrent tumor weight in FGS+NAC was significantly less than BLS+NAC (p = 0.001). The metastatic recurrent weight in the FGS+NAC group compared to the FGS only group was marginally significant (0.059). The average total recurrent tumor weight in FGS only was significantly less than BLS only (p = 0.037), and that in FGS+NAC was also significantly less than BLS+NAC (p = 0.004) (Figures 5 and 6). The recurrence rate of FGS+NAC was also significantly less than BLS+NAC (p = 0.008). FGS+NAC significantly reduced the metastatic recurrence frequency to one of 8 mice compared to FGS only where metastasis recurred in 6 out of 8 mice and BLS+NAC where it occurred in 7 out of 8 mice (p = 0.041) (Table 1).

**Table 1:** Recurrence rate of PDOX in each treatment group.

	local	metastatic
**BLS only**	8/8 (100%)	7/8 (87.5%)
**BLS+NAC**	8/8 (100%)	7/8 (87.5%)
**FGS only**	6/8 (75%)	6/8 (75%)
**FGS+NAC**	6/8 (75%)	1/8 (12.5%)*

* p = 0.041, compared to FGS only.

## Discussion

In a previous study, we conjugated a monoclonal antibody specific for the tumor-associated antigen CA19-9 with the AlexaFluor 488 green fluorophore. We were able to demonstrate in vivo binding of the antibody fluorophore conjugate to the tumor tissue in an orthotopic mouse model of human pancreatic cancer [5]. This fluorescence facilitated differentiation between normal and tumor tissue within the pancreas and also revealed microscopic foci or tumor implants within the spleen, liver, and peritoneum which were not visible under standard light microscopy. This study offered a novel technique to facilitate the intraoperative identification of both primary tumor and small metastatic lesions that may be missed at the time of surgery in those patients whose tumors express the tumor-associated antigen CA19-9.

In another study, we compared a hand-held imaging system with larger imaging systems previously used for FGS [13]. In a PDOX model labeled with Alexa Fluor 488-conjugated anti-CA 19-9 antibody, only the portable hand-held device could distinguish the residual tumor from the background, and complete resection of the residual tumor was achieved under fluorescence navigation, suggesting this system can be applied to the clinic in the near future to enable widespread application of FGS.

There are several novel aspects to the present study that should be emphasized. To the best of our knowledge, this is the first study that has utilized the combination of NAC and a CA 19-9 antibody conjugated fluorophore for FGS of pancreatic cancer. Furthermore, the present study took advantage of a longer wavelength dye, DyLight 650, which we have previous shown has better tissue penetration compared to AlexaFlour 488 [21]. In addition, the PDOX model developed in our laboratory, and used in the present study, allows for individualized therapy that is not available with pancreatic cancer cell line models [7]–[14], [23]. PDOX models can be helpful to determine if an individual’s tumor is sensitive to various NAC regimens. The most novel and unexpected finding was that FGS+NAC eliminated pancreatic cancer metastases in seven out of eight mice.

For bright light surgery, tumors were removed with grossly negative margins under standard bright-field using an MVX10 microscope. For fluorescence-guided surgery, tumor resection was guided by labeling the tumors with an anti-CA 19-9 antibody labeled with a 650 nm fluorophore. The pancreatic cancer PDOX used in this study had a very aggressive behavior. At FGS, we detected some tiny tumors spreading around the primary tumors, which could not be detected under normal macroscopic inspection. At the first surgery, the surgical margin was exterior to the tumor border which was recognized macroscopically. However, a larger margin provided by FGS appears insufficient to lower or prevent metastatic recurrence which required NAC in addition to FGS (Table 1). However, the larger margins afforded by FGS are necessary to lower or prevent metastatic recurrence, as the combination of BLS and NAC are ineffective to lower or prevent metastatic recurrence (Table 1).

All mice in this study were euthanized 90 days after BLS or FGS and therefore, we were not able to compare survival differences between the groups. However, as seen in Table 1, the metastatic recurrence rate in FGS+NAC was significantly less than FGS only (p = 0.041), suggesting that FGS+NAC improves the survival of pancreatic cancer patients compared to FGS only.

In summary, we have determined the efficacy of NAC with GEM in combination with FGS on a pancreatic cancer PDOX model. The results from this study indicate that NAC in combination with FGS can reduce or even eliminate metastatic recurrence of pancreatic cancer sensitive to NAC. This is an important result for the future more effective treatment of pancreatic cancer. The present study further emphasizes the power of the PDOX model which enables metastasis to occur and thereby identify the efficacy of NAC on metastatic recurrence.
